# Increased prevalence of diabetes mellitus and its metabolic risk factors from 2002 to 2017 in Shanghai, China

**DOI:** 10.1111/1753-0407.70003

**Published:** 2024-10-07

**Authors:** Qinping Yang, Jingyan Tian, Yanyun Li, Qinghua Yan, Wenli Xu, Chaowei Fu, Minna Cheng, Yan Shi

**Affiliations:** ^1^ Division of Chronic Noncommunicable Diseases and Injury Shanghai Municipal Center for Disease Control and Prevention Shanghai China; ^2^ Department of Endocrine and Metabolic Diseases, Shanghai Institute of Endocrine and Metabolic Diseases, Ruijin Hospital Shanghai Jiao Tong University School of Medicine Shanghai China; ^3^ Department of Social Medicine, School of Public Health; NHC Key Laboratory of Health Technology Assessment Fudan University Shanghai China

The prevalence of diabetes in China continues to climb at an alarmingly rapid rate relative to other Asian countries.^1^, ^2^, ^3^ And the prevalence in Shanghai was much higher than other areas in China.^4^, ^5^, ^6^ Diabetes has therefore gradually developed into a significant public health concern affecting the health of Shanghai residents. Interventions on high‐risk population of diabetes were very cost‐effective for diabetes prevention supported by strong evidence.^7^ It was shown that metabolic factors such as obesity, hypertension, and dyslipidemia may be risk factors for diabetes.^8^, ^9^, ^10^

>Data sourced from the 2002, 2009, and 2017 Shanghai Diabetes Mellitus Epidemiological Investigation and the 2013 Shanghai Non‐communicable Disease and Risk Factors Surveillance databases were integrated. In these investigations, information on demographic characteristics, lifestyle, personal histories, and family histories of diabetes and other diseases was collected by questionnaires. Heights, weights, waist circumferences, and blood pressures were obtained using a standardized protocol. A venous blood sample was collected from each participant after an overnight fast of at least 10 h in all investigations; for each subject without a history of diabetes, a blood sample was drawn 120 min after an oral glucose tolerance test after imbibing a standard 75‐g glucose solution. Compound Annual Growth Rate (CAGR) = (Ending Value/Beginning Value) ^ (1/n) ‐1, where “n” represents the number of years of observation. Cochran–Armitage trend test was used to analyze the temporal trends. Logistic regression was utilized to estimate the odds ratio (OR). The population attributable risk percentage (PAR%), which can be used to evaluate the possible reduction in the prevalence of diabetes after elimination of risk factors,^11^ was calculated with OR.

From 2002 to 2017, the standardized prevalence rates of diabetes among Shanghai residents aged 35–74 years old rose from 10.14% to 18.47%. The CAGR was 4.08%, which was higher than that for rural southwest China (3.05%),^12^ New York (1.79%),^13^ Thailand (3.54%),^14^ or India (1.65%–3.83%).^15^ The prevalence of diabetes rose over time for all groups but was higher in men, elderly, and urban residents, a phenomenon also observed in other areas in China.^12^, ^16^ The gap of prevalence between men and women gradually widened (CAGR: 4.55% vs. 3.37%), which was similar in Shenzhen (a first‐tier city in China)^16^ (Figure 1). The prevalence of all metabolic risk factors included in this study increased across years (*p*_trend_ <0.05). All four risk factors were positively correlated with diabetes mellitus (*p* < 0.05), but the ORs did not changed significantly (*p*_trend_ >0.05). The PAR% of hypertension and central obesity were higher than other factors. But only the PAR% of hypertension showed an upward trend (*p*_trend_ <0.05), mainly due to its more rapidly increased prevalence (Table 1).

**FIGURE 1:**
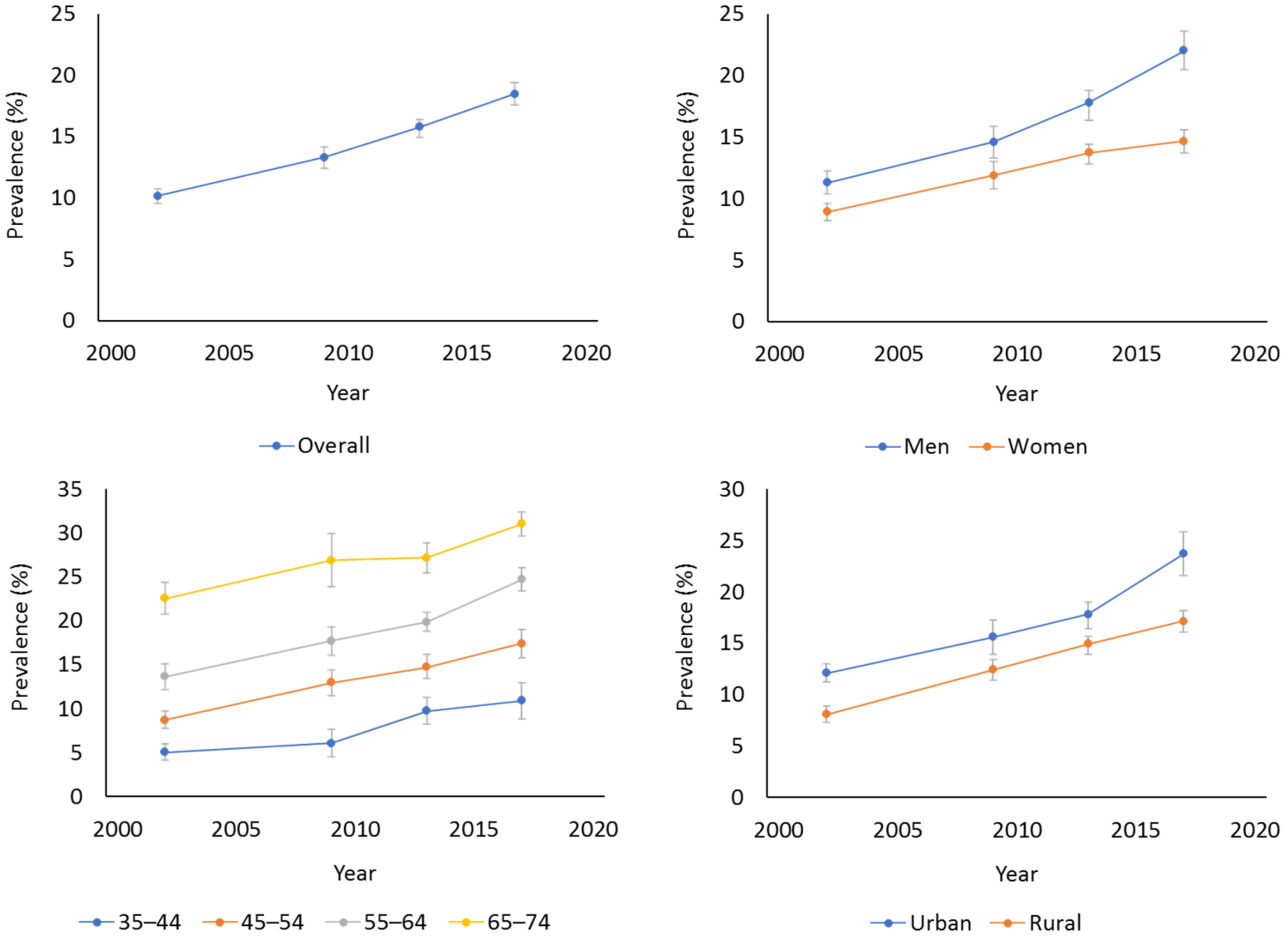
Trends in standardized prevalence of diabetes among Shanghai residents.

**TABLE 1:** Prevalence of different metabolic risk factors prevalence, ORs, and PAR%s associated with diabetes from 2002 to 2017 in Shanghai.

Variable	*%*				*p*_trend_	CAGR (%)	OR (95% CI)^a^				*p*_trend_	PAR%^a^				*p*_trend_
	2002	2009	2013	2017			2002	2009	2013	2017		2002	2009	2013	2017	
Hypertension	27.83 (26.84–28.82)	33.62 (32.26–34.99)	41.53 (40.34–42.72)	51.32 (49.72–52.93)	<0.01	4.16	2.05 (1.59–2.65)	2.45 (1.92–3.12)	2.33 (2.07–2.63)	2.19 (1.98–2.44)	0.63	22.64 (13.61–32.28)	32.76 (22.87–42.65)	35.63 (30.07–41.09)	37.98 (32.65–43.18)	0.02
Dyslipidemia	33.41 (32.25–34.57)	34.26 (32.75–35.77)	45.30 (43.96–46.64)	40.14 (38.61–41.68)	<0.01	1.23	2.37 (1.72–3.27)	2.28 (1.85–2.82)	1.85 (1.67–2.04)	1.95 (1.78–2.14)	0.14	31.46 (18.91–43.98)	30.53 (21.75–39.40)	27.68 (22.67–32.71)	27.69 (23.24–32.21)	0.07
Overweight or obesity	50.47 (49.08–51.86)	50.11 (48.25–51.97)	52.57 (51.09–54.05)	58.94 (57.06–60.82)	<0.01	1.04	1.87 (1.51–2.30)	1.66 (1.38–2.01)	1.71 (1.55–1.89)	1.70 (1.54–1.86)	0.24	30.41 (20.05–40.36)	24.94 (15.43–34.33)	27.21 (21.97–32.43)	29.09 (23.65–34.47)	0.76
Central obesity	42.06 (40.79–43.33)	51.28 (49.42–53.14)	58.79 (57.26–60.33)	63.50 (61.57–65.42)	<0.01	2.78	2.24 (1.79–2.82)	1.94 (1.60–2.34)	1.87 (1.68–2.09)	1.89 (1.70–2.10)	0.09	34.37 (24.28–44.12)	32.41 (22.78–41.65)	33.92 (28.08–39.58)	36.11 (30.24–41.76)	0.56

Abbreviations: CAGR, compound annual growth rate; CI, confidence interval; OR, odds ratio; PAR%, population attributable risk percentage.

^a^ After adjusting for sex, age, region, education years, diabetes mellitus (DM) family history, and lifestyles (never exercising, currently smoking, and current consuming alcohol).

The overall situation with regard to diabetes prevention and treatment in Shanghai is challenging. The prevalence rates of diabetes in various groups rose, but faster in men. Furthermore, attention must be given to the intervention and control of metabolic risk factors such as hypertension that contribute significantly to the prevalence of diabetes. It is crucial to implement continuous registration, screening, and early intervention measures in the high‐risk population for inhibiting or delaying the onset of diabetes.

## CONFLICT OF INTEREST STATEMENT

The authors declare no conflicts of interest.
